# Polyhexanide, Povidone‐Iodine, and Hypochlorous Acid Show High In Vitro Antimicrobial Efficacy Against Pathogens Commonly Associated With Equine Infectious Keratitis

**DOI:** 10.1111/vop.70141

**Published:** 2026-01-19

**Authors:** Leonie Maria Stolle, Hilke Oltmanns, Jessica Meißner, Frederik Heun, Ann‐Kathrin Schieder, Hinrich Tönjes Wolff, Bernhard Ohnesorge, Claudia Busse

**Affiliations:** ^1^ Clinic for Horses University of Veterinary Medicine Hannover, Foundation Hannover Lower Saxony Germany; ^2^ Department of Pharmacology, Toxicology and Pharmacy University of Veterinary Medicine Hannover, Foundation Hannover Lower Saxony Germany; ^3^ LABOKLIN GmbH & Co. KG Bad Kissingen Bavaria Germany; ^4^ Department of Ophthalmology, Clinic for Small Animals University of Veterinary Medicine Hannover, Foundation Hannover Lower Saxony Germany

**Keywords:** antiseptics, bacteria, cornea, eye, horse, ophthalmic infection

## Abstract

**Objective:**

To determine the in vitro antimicrobial activity of specific antiseptics against common equine ocular surface pathogens.

**Methods:**

*Staphylococcus aureus* ( *S. aureus* ) (*n* = 12), *Streptococcus equi* subspecies zooepidemicus (*S. zooepidemicus*) (*n* = 9), *Enterobacter hormaechei* ( *E. hormaechei* ) (*n* = 6), and *Bacillus cereus* ( *B. cereus* ) (*n* = 5) were collected from corneal samples of horses with ulcerative keratitis. Reference strains were included. Minimum bactericidal concentrations (MBC) of polyhexanide, povidone‐iodine, and hypochlorous acid were tested using the microdilution method. After incubation with the antimicrobial agent, the inocula were subcultured according to the Clinical and Laboratory Standards Institute guidelines. Colony growth was manually counted and photographically documented.

**Results:**

The MBC values of polyhexanide were 0.8–3.2 ppm for *S. aureus* , 0.8–1.6 ppm for S. zooepidemicus, 1.6–3.2 ppm for *E. hormaechei* , and 1.6–6.4 ppm for *B. cereus* . For povidone‐iodine, values were 8–32 ppm for *S. aureus* , 4–16 ppm for S. zooepidemicus, 8–16 ppm for *E. hormaechei* , and 8–16 ppm for *B. cereus* . For hypochlorous acid, values were 0.4–6.4 ppm for *S. aureus* , 0.4–3.2 ppm for S. zooepidemicus, 0.8–1.6 ppm for *E. hormaechei* , and 1.6–6.4 ppm for *B. cereus* . The MBC values of methicillin‐resistant *S. aureus* isolates were comparable to those of methicillin‐susceptible isolates.

**Conclusion:**

All antiseptics are highly efficient against common equine ocular bacterial surface pathogens, in concentrations that are well below those of commercially available products. In accordance with the One Health approach, these findings highlight their potential in treating infectious ocular surface disease either as an alternative or alongside topical antibiotics. Further in vivo and clinical studies are required to investigate the translatability of their in vitro effectiveness to clinic cases.

## 1 Introduction

Equine infectious keratitis is a common and potentially vision‐ and globe‐threatening ocular disease that requires intensive management [1, 2, 3, 4]. While some cases of equine keratitis can lead to vision loss or require enucleation, many horses achieve favorable visual outcomes with appropriate treatment [2, 4, 5, 6]. A variety of pathogens can be involved in the development of equine infectious keratitis. In most cases, a positive bacterial culture can be obtained [7, 8, 9]. Considering that one study demonstrated that bacteria were involved in 85% of isolates from infectious keratitis cases, with 55% of cultures yielding bacteria as the sole pathogen, it indicates a significant role of bacterial agents in the disease's etiology [7]. There are geographical differences in the involved bacteria, and data from multiple countries have identified Staphylococcus, Streptococcus, and Pseudomonas as the most common bacterial genera [7, 9, 10, 11, 12, 13, 14]. Alongside bacteria, fungal pathogens also contribute significantly to the disease's pathogenesis in horses [5, 15, 16].

In equine infectious keratitis, early treatment is crucial for preventing complications and preserving vision [1]. Initial treatment focuses on topical antibiotics to manage the infection and support healing [3, 4, 17, 18]. Due to the urgency of starting treatment, initial antimicrobial therapy must be empirical and initiated before culture and sensitivity results are available [3, 18]. Currently, this involves selecting topical antibiotics based on clinical evaluation and cytology examinations to characterize the infection and guide appropriate initial therapy. However, antibiotic susceptibility testing remains essential for guiding treatment, as resistance patterns vary among bacteria [11, 15], and it can facilitate adequate changes in medication if the initial treatment is ineffective in controlling the disease. The prevalence of multidrug‐resistant isolates has risen in recent years, making antimicrobial resistance an escalating concern in clinical practice [9, 10]. This emphasizes the need for careful and judicious use of antibiotics in the treatment of infectious keratitis [19, 20, 21].

The rising resistance, combined with the fact that high concentrations and frequent topical application of certain antibiotics, such as gentamicin, ciprofloxacin, and tobramycin, can delay epithelial regeneration in experimental models [22], underscores the need for alternatives.

In recent years, the EU regulations [23] have increasingly restricted the use of critically important antimicrobials in veterinary medicine, requiring microbiological justification and fostering a more controlled and monitored prescription practice. Furthermore, antagonistic effects between concurrently administered antibiotics and their mutual interactions may reduce overall therapeutic efficacy [24]. All these points underscore the urgent need to explore alternative substances with antimicrobial properties for the treatment of equine infectious keratitis [25]. This is in accordance with the One Health approach [26], which emphasizes the interconnectedness of human, animal, and environmental health in addressing emerging zoonoses and other health challenges [27].

Antiseptics are increasingly being considered as alternatives to antibiotics in various medical fields [28, 29, 30, 31, 32, 33]. While both antibiotics and antiseptics have antibacterial properties, they are distinct categories of agents [34]. Antiseptics demonstrate broader spectrum antimicrobial activity and are less likely to induce resistance compared to antibiotics [35].

Several antiseptics, including povidone‐iodine (PVP‐I), hypochlorous acid (HOCl), and polyhexanide (PHMB), have been investigated for ocular use with positive outcomes to date. PVP‐I remains the main antiseptic agent used for preoperative ocular antisepsis in ophthalmology [29]. However, PHMB shows broad‐spectrum antimicrobial activity against bacteria, most fungi, and protozoa [36, 37] and HOCl has a potent oxidative antimicrobial effect against various pathogens. This may make them promising alternatives [38, 39]. Previous studies have investigated these antiseptic agents for ocular use, demonstrating good efficacy and tolerability for ocular applications [36, 37, 38, 39, 40]. Considering their proven antimicrobial effects and encouraging safety data, PHMB, PVP‐I, and HOCl have promising potential to become essential therapeutic options in the future management of infectious keratitis as effective and well‐tolerated alternatives to conventional antibiotics.

### 1.1 Purpose

The purpose of this study was to determine the in vitro antimicrobial activity of PHMB, PVP‐I, and HOCl against the most common pathogens found in eyes from horses with equine ulcerative bacterial keratitis.

We hypothesized that all tested antiseptics effectively inhibit bacterial growth of the most common bacterial pathogens in equine ulcerative bacterial keratitis at concentrations shown to be tolerated on the ocular surface.

## 2 Materials and Methods

### 2.1 Bacterial Isolates

Between August 2022 and August 2024, swab samples were collected from horses diagnosed with infectious ulcerative keratitis by experienced veterinarians based on clinical examination. Samples were obtained during routine diagnostic and therapeutic procedures. They originated from both cases examined at the authors' institution, the Clinic for Horses of the University of Veterinary Medicine Hannover, Germany, and from external equine clinics and veterinarians in Germany. All samples were processed either at the Institute of Microbiology of the University of Veterinary Medicine Hannover, Germany, or at LABOKLIN GmbH & Co. KG, Bad Kissingen, Germany. Cases were included if a corneal defect with a clear ulcerative appearance (epithelial, stromal, or melting ulcer) was present, while samples originating from outside Germany were excluded. When available, clinical information such as diagnosis, previous antimicrobial treatment, ulcer duration, and sampling site was recorded. Antimicrobial susceptibility results were documented when provided by the respective laboratory. All clinical and microbiological data were compiled and analyzed retrospectively in another project.

The isolates were identified via matrix‐assisted laser desorption/ionization time‐of‐flight mass spectrometry (MALDI‐TOF MS).

These clinical samples were collected and analyzed to determine the most frequently occurring bacterial species. The most prevalent isolates were subsequently included in the antiseptic testing performed in this study. *Pantoea agglomerans* was the most commonly identified species overall, but it was evaluated separately in a previous study [41].

The four isolates with the highest regional incidence that were tested included 12 isolates of *Staphylococcus aureus* ( *S. aureus* ), nine isolates of *Streptococcus equi* subspecies zooepidemicus (*S. zooepidemicus*), six isolates of *Bacillus cereus* ( *B. cereus* ), and five isolates of *Enterobacter hormaechei* ( *E. hormaechei* ). Six of the *S. aureus* isolates were methicillin‐resistant.

Additionally, reference strains from the German Collection of Microorganisms and Cell Cultures (DSMZ, Braunschweig, Germany) were utilized, including *S. aureus* (DSM 1104), S. zooepidemicus (DSM 20727), and *B. cereus* (DSM 31).

### 2.2 Antiseptic Agents

Three antiseptic agents, including PHMB, PVP‐I, and HOCl, were evaluated and tested against all bacterial isolates, with all dilutions freshly prepared on the day of testing (Table 1).

**TABLE 1:** Overview of the antiseptic agents used in this study, including the commercially available products, their original concentrations, and the dilution ranges obtained by dilution with phosphate‐buffered saline.

Antiseptic agent	Used a commercially available product	Product concentration	Experimental model dilutions
PHMB	Polyhexanid‐Lösung, Fagron, Glinde, Germany	20% =200 000 ppm	0.000005%–0.00256% =0.05–25.6 ppm
PVP‐I	PVP‐I 1 −100 g Poly(vinylpyrrolidone)‐iodine complex PVP‐I, Povadyne antiseptic, SIGMA‐ALDRICH CHEMIE GmbH, Steinheim, Germany	100% =1 000 000 ppm	0.00005%–0.0256% =0.5–256 ppm
HOCl	Vetericyn.VF + plus eye and ear solution, Ecuphar GmbH, Greifswald, Germany	0.0275% =275 ppm	0.000005%–0.00256% =0.05–25.6 ppm

*Note:* Although these concentrations were generated as part of the experimental dilution protocol, only the concentrations listed in Table 2 were finally tested.

### 2.3 Preservation of Bacterial Isolates

Bacterial isolates were preserved by initial cultivation in Lysogeny broth (NaCl, 5 g/L; yeast extract, 5 g/L; tryptone, 10 g/L) overnight. Freshly isolated strains were suspended in 80% glycerol stock solution and stored in cryovials at −70°C. Before carrying out the experiment, fractional streaks were cultured on 5% Columbia Sheep Blood Agar (COLUMBIA AGAR WITH SHEEP BLOOD, Oxoid Deutschland GmbH, Wesel, Germany) and incubated for 16–24 h at 37°C to ensure viable cultures.

### 2.4 Determination of Minimum Bactericidal Concentrations (MBCs)

The antimicrobial susceptibility of each bacterial isolate was assessed according to the Clinical and Laboratory Standards Institute (CLSI) guidelines for determining the bactericidal activity of antimicrobial agents (M26‐A) [42]. A previously established modification of the CLSI M26‐A [43] method was used for MBC testing. The MBC was assessed using a subculture method on agar plates after the bacteria had been incubated with various concentrations (Table 2) of the antimicrobial agent.

**TABLE 2:** Concentration of used antiseptics in each dilution stage (V1–V8).

	V1	V2	V3	V4	V5	V6	V7	V8
PHMB	6.4 ppm	3.2 ppm	1.6 ppm	0.8 ppm	0.4 ppm	0.2 ppm	0.1 ppm	0.05 ppm
PVP‐I	64 ppm	32 ppm	16 ppm	8 ppm	4 ppm	2 ppm	1 ppm	0.5 ppm
HOCl	6.4 ppm	3.2 ppm	1.6 ppm	0.8 ppm	0.4 ppm	0.2 ppm	0.1 ppm	0.05 ppm

In this study, the MBC is defined as the lowest concentration at which 99.9% of the original bacterial population in the final inoculum is killed, as previously described [41, 42, 43].

Each isolate was tested in triplicate. Bacterial suspensions were adjusted in sterile saline (NaCl 0.9%, BRAUN, Frankfurt am Main, Germany) to 0.5 McFarland units (approximately 1.5 × 10^8^ CFU/mL) using a densitometer (Biosan, Riga, Lettland). Further, the bacterial suspension was diluted twice in a 1:9 ratio to achieve an approximate concentration of 1.5 x 10^6^ CFU/mL for the bacterial suspension.

The sterile antiseptics (PHMB, PVP‐I, HOCl) were diluted in eight stages in a 1:1 ratio with phosphate‐buffered saline (PBS) (Table 2). Each experimental setup included positive and negative controls.

A volume of 0.25 mL of each antiseptic dilution was combined with an equal volume of bacterial inoculum/suspension, which was diluted to a final concentration of approximately 1.5 × 10^6^ CFU/mL, and incubated for 10 min. Following incubation, 0.1 mL of the mixture was combined with 0.9 mL of Dey‐Engley‐neutralizing broth (Merck KGaA, Darmstadt, Germany) to terminate the antiseptic action, as used in a previous study [43], followed by an additional 5‐min incubation. The neutralized mixture was then plated onto 5% Columbia sheep blood agar plates and incubated at 37°C for 16–20 h. Following this protocol, 7500 colony‐forming units (CFU) were expected to be plated on each agar plate. The negative control contained no bacterial suspension but was handled in the same manner as all other samples (neutralizer and equal volumes). Similarly, the positive control did not contain any antiseptics, but the same amount of bacterial suspension as the other samples.

At the beginning of the experiment, preliminary tests were performed to verify that the densitometer‐based adjustment in McFarland units [45, 46], corresponded to the targeted initial CFU per isolate. For species in which the desired concentration could not be reliably achieved using the McFarland setting, additional procedures were implemented, notably for *Bacillus cereus* , as described in the corresponding section.

The bacterial concentrations used for all analyses were therefore based on the previously validated measurements. Approximately 7500 CFU were applied per plate. Colony counts were recorded only for plates containing up to 300 CFU, whereas higher counts were classified as too numerous to count, in accordance with standard microbiological guidelines, as overcrowding prevents accurate enumeration [44, 47].

**FIGURE 1:**
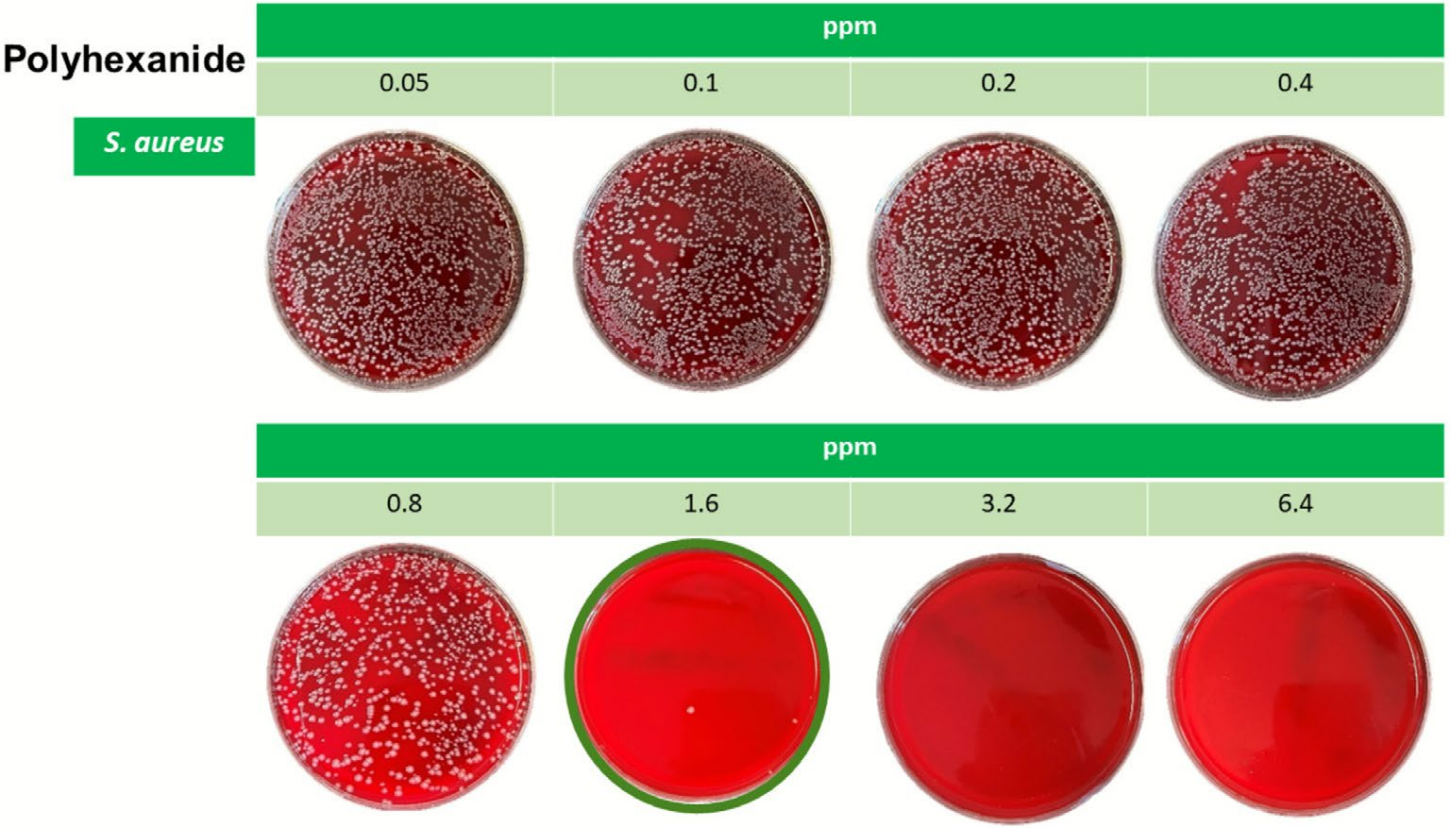
This figure illustrates an example of the MBC assessment of PHMB against a *S. aureus* isolate. The image displays eight agar plates corresponding to serial dilutions of PHMB, with concentrations indicated in ppm above each plate. Following incubation (16–20 h), bacterial growth was visually assessed. Each plate was inoculated with approximately 7500 CFU of the test strain. In this specific example, visible growth of 4 CFU was observed at a PHMB concentration of 1.6 ppm and none at higher concentrations, indicating a ≥ 99.9% reduction in viable bacterial count, thus defining the MBC at 1.6 ppm for this isolate.

Bacterial growth exceeding this threshold was therefore evaluated visually by counting after an incubation time of 16–20 h, with the critical number of colonies set at 7 CFU/agar, indicating a 99.9% inhibition of bacterial growth, based on an expected growth of approximately 7500 CFU per plate in the absence of antimicrobial activity (Figure 1).

The incubation period was narrowed to 16–20 h to improve accuracy by preventing overgrowth and allowing clearer counts, which was not necessary for pure culture growth but essential for antiseptic testing.

The MBC values were determined by counting the bacterial colonies grown on agar plates and performing calculations based on these counts.

### 2.5 Bacillus cereus

Initial testing of *B. cereus* isolates revealed insufficient growth in pretests and positive control samples, as indicated by a reduced number of colonies and a spread‐out growth pattern despite the absence of antiseptic agents. This finding suggests that adjusting the bacterial suspension solely to 0.5 McFarland units using a densitometer was not appropriate for this species. Due to the irregular colony formation, counting and defining MBC values were unreliable. Therefore, a preliminary growth assessment was conducted to ensure accurate determination.

Six *B. cereus* suspensions were prepared, and triplicate plating was performed at inoculation levels of 7500, 750, and 75 CFU per plate. The actual growth of colonies was counted to calculate a correction factor for the bacterial suspension dilution, allowing for the precise determination of MBCs in subsequent assays. Following this factor, the bacterial suspension with a theoretical concentration of 0.5 MFU (of 1.5 × 10^8^ CFU/mL) was only diluted in a 1:1.6 ratio to achieve a realistic bacterial concentration of 1.5 × 10^6^ CFU/mL.

The main experiment was then performed as described above.

### 2.6 Data Analysis

MBC values were determined by calculating viable bacterial reductions based on an expected untreated growth level of 7500 CFU per plate. The minimum bactericidal concentration was defined as the lowest antiseptic concentration producing a ≥ 99.9% reduction in colony‐forming units (≤ 7 CFU per plate). The corresponding concentration state is considered the MBC.

A descriptive analysis of the collected data was performed using Excel (Version 2405, June 11, Microsoft Office Professional). The figures were created with Excel, Microsoft PowerPoint 2016, and GraphPad Prism 9 (GraphPad Software Inc.).

## 3 Results

All tested antiseptics showed an in vitro antimicrobial effect at specific concentrations. None of the isolates exhibited resistance.

Positive controls consistently showed the expected bacterial growth, which was confirmed visually, validating the functionality of the culture media and methodology. Conversely, the negative controls consistently showed no growth, indicating successful prevention of contamination during the procedures.

### 3.1 Polyhexanide

**FIGURE 2:**
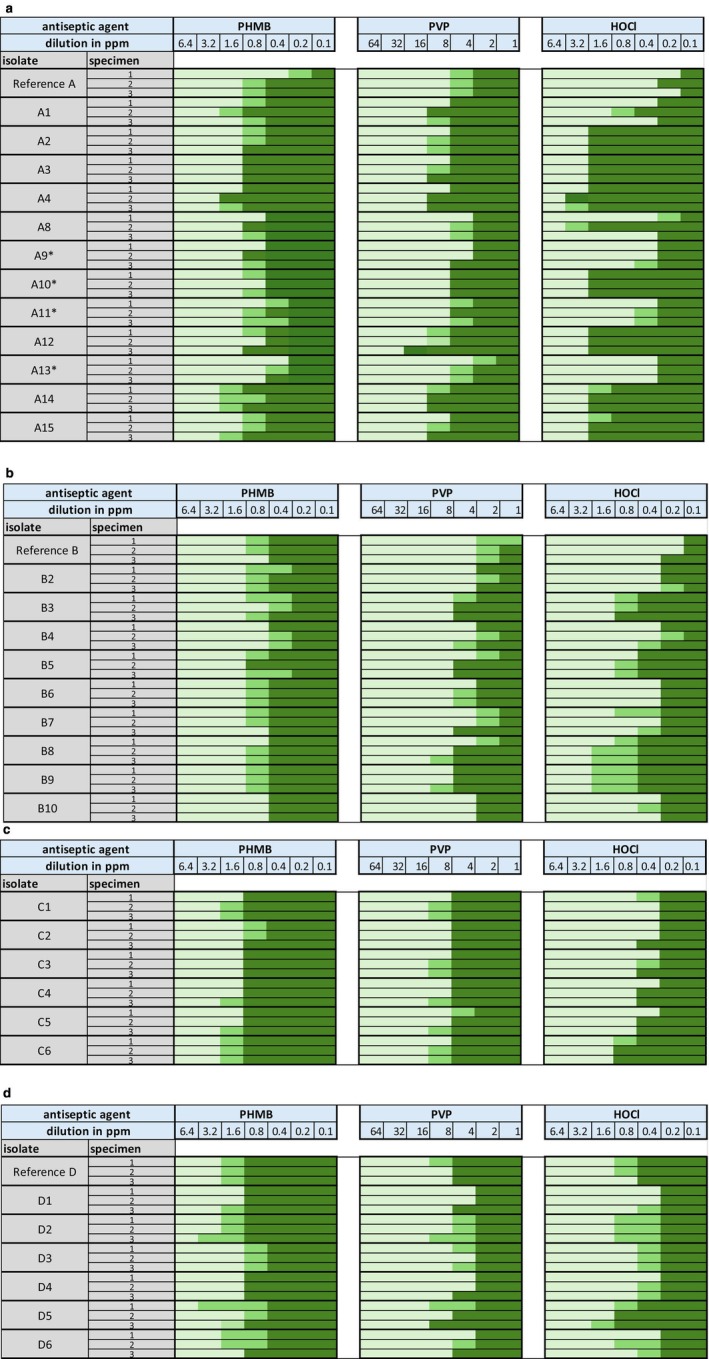
Results for all tested isolates of the four bacteria species against the three antiseptics. Each isolate was tested in triplicate (row 1–3). Dark green fields indicate no observable reduction in colony growth with colony counts > 300. Medium green fields indicate a reduction of colony growth (colony counts between 8 and 300) but do not show a reduction that matches the MBC definition (≤ 99.9%). Light green fields indicate a reduction in bacterial growth of ≥ 99.9%, which corresponds to the MBC (0–7 colonies). (a) Results for *S. aureus* , (b) Results for *S. zooepidemicus*, (c) *Results for E. hormaechei*, and (d) Results for *B. cereus* . HOCl = hypochlorous acid, PHMB = polyhexanide, PVP‐I = povidon iodine, Reference = reference strain (DSMZ). * = methicillin resistant species.

For all isolates the MBC for polyhexanide ranged from 0.8 to 6.4 ppm. For *S. aureus* , MBC values across all isolates ranged from 0.8 to 3.2 ppm (Figure 1). For the methicillin‐resistant isolates it ranged from 0.8 to 1.6 ppm and for the methicillin‐susceptible from 1.6 to 3.2 ppm. For *S. zooepidemicus*, the MBC was 1.6 ppm for nearly all isolates, with one isolate showing a lower value of 0.8 ppm. For *E. hormaechei* , MBC values ranged between 1.6 and 3.2 ppm. For *B. cereus* , MBC values ranged from 1.6 to 6.4 ppm (Figure 2a–d).

### 3.2 Povidone‐Iodine

For all isolates the MBC for povidone‐iodine ranged from 8 to 32 ppm. The MBC values for PVP‐I ranged for *S. aureus* from 8 to 32 ppm, with an MBC for the methicillin‐resistant isolates of 8 ppm and for the methicillin‐susceptible values from 8 to 32 ppm. For *S. zooepidemicus*, it ranged from 4 to 16 ppm. For *E. hormaechei* , the MBC was 16 ppm for most isolates, with one isolate showing a lower value of 8 ppm. For *B. cereus* , the values ranged between 8 and 16 ppm (Figure 2a–d).

### 3.3 Hypochlorous Acid

For all isolates the MBC for hypochlorous acid ranged from 0.4 to 6.4 ppm. For *S. aureus* , the MBC values ranged from 0.4 to 6.4 ppm. For the methicillin‐resistant isolates it ranged from 0.4 to 3.2 ppm and for the methicillin‐susceptible from 0.4 to 6.4 ppm. For *S. zooepidemicus*, the MBC values ranged from 0.4 to 3.2 ppm. For *E. hormaechei* , most isolates required an MBC of 0.8 ppm, although one isolate required 1.6 ppm. For *B. cereus* , the MBC values ranged between 1.6 and 6.4 ppm (Figure 2a–d).

The MBC values for methicillin‐resistant *S. aureus* were not higher than for methicillin‐susceptible ones.

Across all isolates and antiseptics, the MBC values fell within a comparable overall range, and no consistent pattern indicating higher or lower requirements for any species or antiseptic was apparent. However, evaluation of Figure 2a–d shows that intraspecies variation was considerable, particularly for hypochlorous acid, with values for *S. aureus* ranging from 0.4 to 64 ppm and within *S. zooepidemicus* from 0.4 to 3.2 ppm. Variation was also observed for povidone‐iodine, with MBCs for *S. aureus* ranging intraspecies from 8 to 32 ppm.

In total, the MBC at which all tested isolates were reliably killed was 6.4 ppm for polyhexanide, 32 ppm for povidone‐iodine, and 6.4 ppm for hypochlorous acid.

## 4 Discussion

In this study, we evaluated the in vitro bactericidal efficacy of PHMB, PVP‐I, and HOCl against clinically relevant pathogens that were isolated from horses with ulcerative keratitis.

All tested antiseptics demonstrated excellent bactericidal activity. The maximal observed MBCs were 6.4 ppm for both PHMB and HOCl, and 32 ppm for PVP‐I. These findings are consistent with previously published data. For PHMB, reported minimal inhibitory concentrations in human isolates range from 0.1 to 25 ppm [48, 49]. The MBC for PVP‐I in this study was 32 ppm, which is consistent with values reported in previous in vitro studies involving other bacterial species [50]. In the case of HOCl, the MBC was 6.4 ppm, notably lower than concentrations reported in earlier studies, where up to 150 ppm was required to achieve bactericidal activity against *S. aureus* [51].

A comparative analysis with a parallel study performed under identical laboratory conditions, involving canine ocular isolates, yielded closely aligned results. In that study, the maximal MBCs were 3.2 ppm for PHMB, 32 ppm for PVP‐I, and 1.6 ppm for HOCl [43]. The similarity of results underlines the consistency and potential cross‐species applicability of these antiseptics, suggesting only a slight species‐specific variance in susceptibility against the agents.

Given the frequent involvement of fungi in infectious keratitis, it is notable that all three antiseptics have also demonstrated strong antifungal activity in other studies [14, 52, 53, 54, 55, 56, 57].

Previous studies have not described differences in susceptibility between methicillin‐resistant and methicillin‐susceptible *S. aureus* [49]. In comparison, our data appear to show a similar pattern.

To evaluate the relevance of our findings, all isolates were compared to reference strains under identical experimental conditions. The comparative analysis revealed no obvious differences between the isolates and the reference strains in any of the antiseptics used.

Several studies have highlighted geographic variation in the composition of the equine ocular microbiota, with Staphylococcus spp., Streptococcus spp., and Pseudomonas spp., as well as other bacteria and fungal organisms, being the most frequently isolated pathogens, depending on the region and study. Recent data indicate a possible increase in Staphylococcus spp. prevalence [7, 10, 11, 12, 15, 19].

We tested the four most frequently detected bacteria of our dataset: *S. aureus* , S. zooepidemicus, *E. hormaechei* , and *B. cereus* . The high prevalence of Staphylococcus ssp. and Streptococcus ssp. is consistent with findings in other countries and confirms their role as the most commonly isolated ocular pathogens across various studies and regions [9, 10, 11, 12, 13, 16].

Variations in the distribution of less dominant genera, potentially driven by geographic and environmental factors, further underscore regional differentiation.

### 4.1 Polyhexanide

PHMB has a broad‐spectrum antimicrobial activity, which involves blocking DNA replication and exerting intracellular effects [58, 59, 60, 61]. This may explain its efficacy and low resistance development. Consequently, bacterial resistance to PHMB has not been reported yet [59, 61]. Several studies show that PHMB has effective antibacterial activity in low concentrations (≥ 2 μg/mL) [56, 63, 64], even against MRSA [59, 65]. PHMB disrupts biofilms [28], promotes re‐epithelialization, and reduces bacterial burden in wounds at well‐tolerated low concentrations [32, 66, 67]. However, these results are derived from wound studies and do not directly reflect corneal conditions.

In human ophthalmology, PHMB has been used at concentrations ranging from 0.001% to 0.02% in ophthalmic formulations and contact lens solutions for the treatment of Acanthamoeba keratitis and as a presurgical antiseptic, demonstrating good tolerability [36, 37, 48, 59, 68]. Except for a sole report with mild adverse effects observed under PHMB 0.08% treatment, this concentration was overall well tolerated [69]. Additionally, an ex vivo study using porcine corneas treated with 0.04% PHMB showed no adverse effects on epithelial integrity or corneal wound healing [70] and therefore provided support for the ocular surface compatibility of PHMB.

While much of the evidence regarding PHMB derives from wound studies [66] whose findings cannot be fully extrapolated to the avascular corneal environment, current evidence supports the safe use of PHMB at low concentrations, demonstrates good tolerability, and suggests potential benefits for antimicrobial benefit.

Notably, our findings reveal a substantial safety margin between the maximum MBC identified in our study (0.00064%) and the concentrations commonly used in clinical studies.

### 4.2 Povidone‐Iodine

PVP‐I is widely used in both human and *Veterinary Ophthalmology* [29, 42, 50, 71, 72] due to its antimicrobial efficacy, antibiofilm activity, and lack of resistance development [66, 71].

PVP‐I exhibits rapid and broad‐spectrum antimicrobial activity [73, 74] by releasing free iodine that penetrates microorganisms and induces oxidative damage to essential proteins, nucleotides, and fatty acids, ultimately leading to cell death [22, 75, 76, 77, 78, 79].

Interestingly, lower concentrations of PVP‐I increase the availability of free iodine and result in enhanced antimicrobial activity until a critical dilution is reached. This effect is explained by the characteristic dilution curve of PVP‐I. As a 10% solution is diluted, the amount of free iodine initially rises and reaches its peak at roughly 0.1%, after which it begins to decline again [79, 80, 81]. Consequently, moderately diluted solutions can exhibit stronger antimicrobial effects than more concentrated formulations. Based on these findings, a concentration of approximately 0.1% appears to be the most suitable for application.

This concentration‐dependent effect is relevant for ophthalmic use in horses, where diluted formulations are commonly employed to balance antimicrobial efficacy with ocular surface tolerability [71, 82]. However, studies show that lower concentrations (0.5%–1%) are generally well tolerated in ophthalmic use [37, 38, 40, 69, 83].

In a study on a rabbit model, repeated application of PVP‐I was used to assess ocular tolerance. The 0.33% solution was well tolerated with no observable side effects, while the 0.5% concentration caused increased conjunctival irritation and delayed epithelial healing by about 1 day [38].

Further adverse effects, such as suspected dry eye syndrome caused by PVP‐I in 5%, have been reported [84], but only at concentrations far exceeding those found to have an antimicrobial effect in our study, considering our maximum observed MBC of 0.0032%. In a recent review, none of the included studies reported any safety concerns with topical PVP‐I [85].

Available animal data indicate that dilute topical PVP‐I is generally well tolerated on the ocular surface when exposure time and concentration are controlled [38, 40, 83]. However, true long‐term safety data from continuous use in animals are currently lacking. Still, small sample sizes and heterogeneity in study design, populations, outcome measures, and PVP‐I concentrations limit current evidence. Therefore, further equine studies with larger sample sizes and standardized PVP‐I concentrations, durations, and outcome measures are recommended to confirm the efficacy of PVP‐I in treating infectious keratitis in horses [85].

### 4.3 Hypochlorous Acid

HOCl is a halogen‐releasing agent, which is most effective at pH 4–7, where the active HOCl form dominates. It also occurs naturally as part of the innate immune response, produced by neutrophils during the oxidative burst [57].

Although its exact mechanism is not fully understood, HOCl acts as a potent oxidant that disrupts microbial cells by damaging key structures and interfering with DNA, protein, and ATP synthesis [75].

HOCl effectively reduces the bacterial load on the ocular surface without altering microbial diversity [86], and it is effective in reducing ocular bacterial load in a concentration of 0.01% without irritation [39].

Experimentally topical HOCl in a concentration of 0.009% reduced pain, infection, and healing time of corneal lesions in experimentally induced infectious bovine keratoconjunctivitis [87].

Furthermore, HOCl had dose‐dependent, favorable effects on fibroblast and keratinocyte migration, making it an ideal wound care agent [88].

Interestingly, among the three antiseptics, only hypochlorous acid is currently available as a commercially approved ocular rinsing solution in the EU (Vetericyn, VF + plus eye and ear solution, Ecuphar GmbH).

When comparing the existing body of evidence on HOCl with our findings, which showed a maximum MBC of 0.00064%, it becomes evident that the concentrations already used in ocular applications are several times higher than the bactericidal levels identified in our study, supporting the classification of such use as safe. These findings, together with existing clinical and experimental data, support the safe and effective use of hypochlorous acid in ophthalmic applications, particularly given the substantial safety margin between its bactericidal threshold and concentrations already in commercial use.

Compared to PVP‐I and PHMB, HOCl offers a wider safety margin, with effective concentrations well above its MBC and no reported irritation. While PVP‐I and PHMB show dose‐dependent side effects, HOCl appears better tolerated, though long‐term data in equine eyes are still lacking.

Povidone‐iodine, polyhexanide, and hypochlorous acid have shown broad‐spectrum efficacy, low resistance potential, and good ocular tolerability at clinically relevant concentrations. Although the concentrations evaluated in this study may not accurately reflect the final concentrations on the ocular surface, the findings offer a positive outlook for their future clinical application.

### 4.4 Clinical Applicability

One of the key challenges is the contact time between the antiseptic agents and the pathogens. In our controlled in vitro setting, we used a defined exposure duration, with neutralization precisely timed at 10 min. In vivo, especially on the equine ocular surface, such consistent and prolonged contact is difficult to achieve due to natural factors, such as the tear film, blinking, and washout rate.

The mean tear volume of a healthy ophthalmological horse is approximately 233.74 μL, with a turnover rate of 13.21%/min and a flow rate of 33.62 μL/min, resulting in a time of complete tear recycling of approximately 7 min [89].

Considering the average volume of a single eye drop, which is approximately 39 μL [90], achieving the desired therapeutic concentration on the ocular surface would require either using at least 7 times the MBC concentration of the antiseptic within a single‐drop formulation, administering a larger volume through multiple drops at a lower concentration, or applying continuous lavage. Given that the average conjunctival sac capacity in horses is approximately 230 μL [91], the administration of 5–6 drops may be appropriate. But it should be noted that the concentration declines rapidly after instillation, and it is difficult to terminate the final contact time even after a defined period, making it difficult to control the antiseptic effect.

This makes the contact time used in our laboratory unrealistic under clinical conditions, unless the eye is flushed with the agent for 10 min. While this might be achievable with a subpalpebral lavage system and manual injection or an infusion pump, it would be highly labor‐intensive and may not be feasible in some clinical settings. Nonetheless, killing times of different concentrations are likely to be well below 10 min for higher concentrations, as shown for Pantoea agglomerans [41].

Consequently, a significantly higher concentration than the MBC determined in our study would be required in clinical patients. However, given the substantial safety margin established in previous studies [36, 37, 38, 39, 68, 83, 87] using much higher concentrations on the ocular surface, there is little concern that such an adjustment would approach toxic levels.

The physiological pH of the healthy equine tear film is approximately 7.84 [92]. In addition, the mean tear film osmolality has been reported at 283.51 mmol/kg, along with specific electrolyte concentrations [89]. However, in eyes affected by infectious keratitis, these values may vary. For example, elevated levels of matrix metalloproteinases and neutrophil elastase have been observed in horses with ulcerative keratitis [93, 94, 95]. As the pH of the ocular surface can influence the efficacy of antiseptics, a shift toward less favorable pH ranges may reduce their antimicrobial activity [96]. The effectiveness of PHMB decreases at lower pH values [97]. In contrast, the activity of PVP‐I is reduced at higher pH [97], likely due to improved solubility of iodine‐releasing metal ions in acidic conditions [98]. The antimicrobial effect of HOCl is also pH‐dependent, with optimal activity between pH 6.0 and 7.5 [99]. A possible strategy to counteract this would be to buffer the antiseptic solution to stabilize pH, to identify a concentration that remains effective despite a potential loss of activity under suboptimal pH conditions, or to adjust the ocular surface pH, for example, through repeated application or controlled flushing via continuous lavage systems.

An essential factor to consider in vivo is the protein error. The protein error refers to the fact that the presence of proteins, particularly albumin, can decrease the effectiveness of antiseptics against bacteria [100]. The total protein concentration in the lacrimal fluid of healthy eyes ranges from 7.0 to 19.5 mg/mL. Serum albumin concentrations in the tear fluid of healthy eyes range from 71.1 to 711.3 μg/mL, making up approximately 1.6% of the total protein content. Eyes affected by ocular disease show significantly higher serum albumin levels in the tear film (median: 679.6 μg/mL) compared to contralateral unaffected eyes (130.0 μg/mL) and reference eyes (200.7 μg/mL; *p* ≤ 0.001) [101]. While total protein content does not differ significantly between healthy and diseased eyes, albumin concentration clearly increases and may even serve as a biomarker of ocular inflammation.

A protein error has been observed with various agents. The antibacterial activity of PHMB‐based antiseptics is significantly diminished in the presence of albumin, especially against *S. aureus* and MRSA [100]. For HOCl, the presence of organic material and inorganic ions leads to faster consumption and loss of antimicrobial activity [102, 103]. Diluted formulations of PVP‐I, such as 0.66% or 1% solutions, have shown reduced antimicrobial efficacy in the presence of proteins, indicating susceptibility to protein interference. Conversely, standard 10% PVP‐I solutions maintain full antimicrobial activity even under high protein loads, suggesting that PVP‐I is not significantly affected by protein errors at very high concentrations [104].

Wolff et al. examined the efficacy of the same antiseptics tested in our study under protein‐free conditions (PBS) and in a protein‐rich medium (CAMHB, approximately 2% protein content). They demonstrated that all three antiseptics were markedly less effective in the presence of protein, requiring substantially higher concentrations to achieve the bactericidal activity. For PVP‐I, a 100‐fold increase in concentration was necessary, while HOCl showed no measurable bactericidal effect in the protein‐rich environment [43].

The reduced efficacy of antiseptics in protein‐rich environments must be considered when applied to the ocular surface, where tear film components may limit antimicrobial activity. Therefore, effective in vitro concentrations may not directly translate to in vivo conditions and should be carefully re‐evaluated.

Given that Wolff et al. [43] observed, for example, a 100‐fold increase in required PVP‐I concentration at 2% protein, a similar effect may be expected on the ocular surface, where albumin levels are approximately 1.6%. Thus, the in vitro MBCs may underestimate the effective in vivo concentration and require adjustment accordingly. Further experimental studies are necessary to determine the exact concentration at which each agent remains effective on the ocular surface with and without discharge.

Another important aspect is the activity against bacterial biofilms. Previous studies have shown that all three antiseptics, PHMB [59, 105], PVP‐I [106, 107], and HOCl [108, 109], are effective in disrupting or preventing biofilm formation. However, these observations are based on in vitro or non‐ocular models, and none have investigated the disruption of biofilms on the corneal surface. This is a relevant limitation, as the cornea represents a special environment that differs fundamentally from vascularized wound tissues. Given that many bacterial species associated with infectious keratitis, including the four isolates examined in our study, are known biofilm producers [109, 110, 111, 112, 113], this gap warrants consideration, but offers a promising outlook that such effects could extend to the ocular surface.

However, biofilm‐associated bacteria exhibit increased resistance to antimicrobials, requiring higher concentrations and longer exposure times for effective eradication, an effect that is likely to also occur with antiseptic susceptibility [114, 115, 116].

There are commercial products licensed for use on the ocular surface, and concentrations already used in other studies indicate that the MBCs we found are significantly lower than the concentrations that can actually be used in practice. This means there is a wide safety margin for adjusting concentrations, especially considering the limitations of in vivo conditions.

**FIGURE 3:**
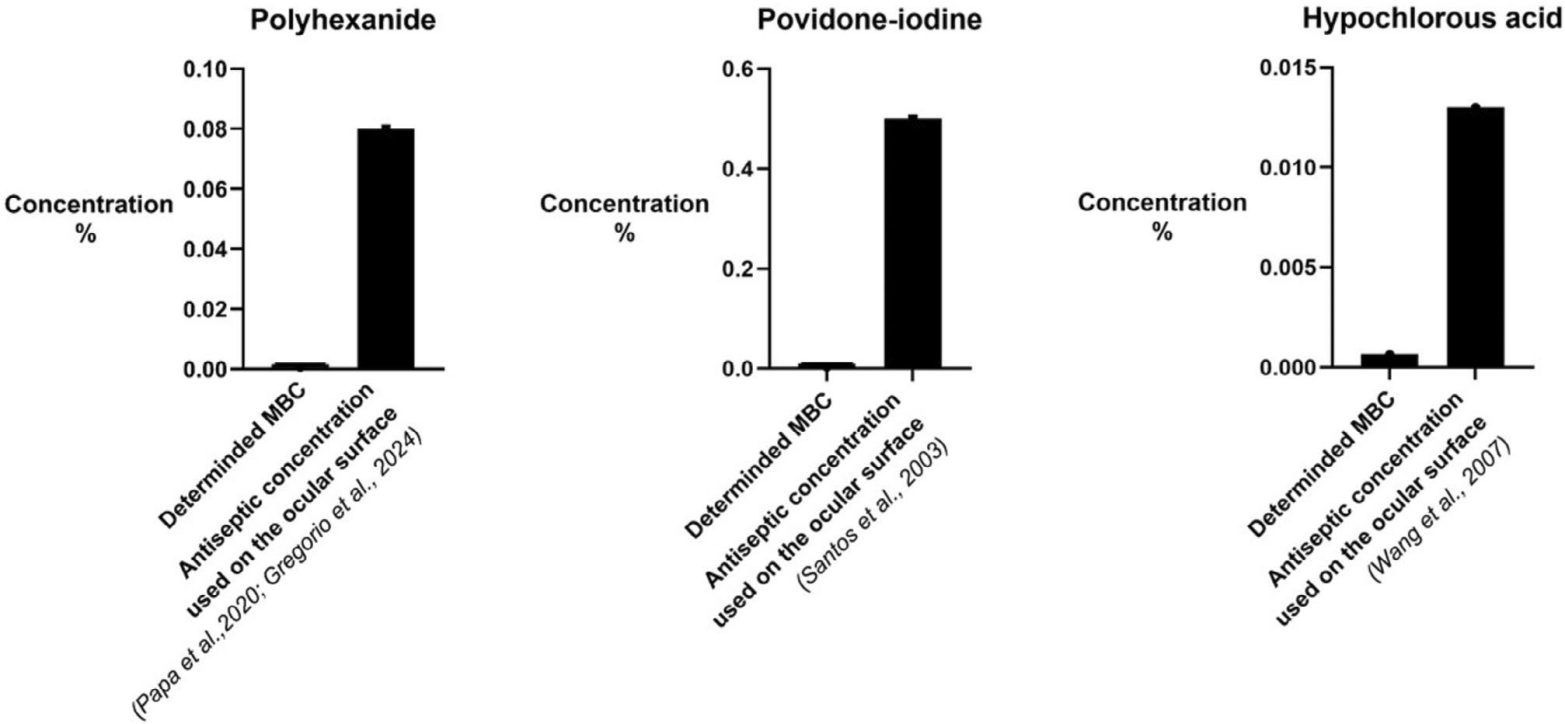
Comparison between the detected MBC concentrations (left bar) and the concentrations that have already been used on the ocular surface (right bar).

A comparison of our MBC values for the different antiseptics with commercial products, as shown in Figure 3, highlights this fact.

For PHMB, assuming a tested concentration of 0.08% or a commercial product at 0.04% (LAVANID 1/2, SERAG WIESSNER GmbH & Co. KG, Naila (Oberfranken), Germany), the usable concentration is up to 62–125 times higher than our MBC.

For HOCl, the commercial product contains a concentration 43 times higher than the MBC we found (Vetericyn, VF + plus eye and ear solution, Ecuphar GmbH, Greifswald, Germany).

And for PVP‐I, typically used at 0.5%, the concentration [117] is 156 times higher than our MBC.

### 4.5 Limitations

One of the main limitations of this study's clinical translation is that it was conducted entirely in vitro, without attempts to simulate key challenges of the ocular surface. Consequently, the results obtained do not reflect the complexity of the in vivo environment, with numerous factors, as discussed in the section on clinical applicability. These aspects were not assessed in the present study and must be considered when interpreting the clinical relevance of the findings.

Another limitation is the relatively small number of bacterial isolates. Although samples originated from different patients and multiple facilities across Germany, most were submitted by two referral clinics and were mainly from the northern regions, resulting in an uneven geographic distribution. The isolates nevertheless showed highly similar susceptibility patterns, supporting the internal consistency of the findings. Even so, a larger and more regionally diverse dataset would be required to strengthen the generalizability of these results, particularly given reported variability in susceptibility among Enterobacteriaceae species [118].

In this study, each experiment involved only one type of bacteria at a time. In real‐life situations, infections often involve more than one pathogen, which may also form biofilms, and such settings can exhibit different behaviors and treatment responses. As our experiments were limited to single‐bacterium models, the findings can currently only be applied to such scenarios. Further studies are needed to determine the efficacy in more complex, polymicrobial or biofilm‐associated infections.

Also, all bacteria included in the study were from a specific geographical region. Since the types of bacteria and their resistance patterns can vary by region [9, 10, 11, 12, 13, 16], the findings may not be universally applicable. However, due to the generally low levels of antiseptic resistance [59, 62, 66, 71], significant differences from findings in other regions are not expected.

Another important consideration is the surface‐restricted activity of topically applied antiseptics. Although all three agents showed excellent in vitro efficacy, their penetration into the corneal stroma is limited [119, 120]. In cases where bacteria are located deeper within the stroma, such as melting ulcers or advanced stromal infections, these pathogens may remain beyond the effective reach of antiseptics.

A further limitation concerns the growth behavior of *Bacillus cereus* . During the experiments, this species exhibited inconsistent colony formation in the positive controls, necessitating an additional correction step to determine the MBCs accurately. Although this issue was most evident in *B. cereus* , it highlights a more general challenge: that bacteria differ in their growth dynamics, and their viable counts at the time of testing do not always perfectly match the calculated inoculum. Such variability can arise from biological differences between strains as well as subtle variations in culture conditions.

The absence of contact‐time experiments represents a limitation of the study. Although one study [41] demonstrated very rapid bactericidal activity of PHMB, PVP‐I, and HOCl against *Pantoea agglomerans* , these findings cannot be directly extrapolated to the broader range of bacterial species included here. Determining killing times for these additional pathogens remains an essential objective for future research.

## 5 Conclusion

Our findings highlight the potential of polyhexanide, povidone‐iodine, and hypochlorous acid in the treatment of infectious ocular surface diseases in horses, either as alternatives to or in combination with topical antibiotics. However, their use as sole agents is likely confined to uncomplicated ulcers, such as superficial ulcers suspected to be contaminated but without established infection, whereas complex ulcers, defined as deep and/or infected ulcers, are more likely to benefit from their use as adjunctive therapies.

Given their antimicrobial activity and the current knowledge of their safety profiles, these agents represent promising candidates for future therapeutic strategies in the management of ulcerative keratitis and may offer effective and well‐tolerated options beyond conventional antibiotic therapy. And in this, they may help to reduce the use of antibiotics in accordance with the One Health approach. Our findings indicate that the effective concentrations of the tested antiseptics were largely consistent across different bacterial species. This uniformity suggests that empirical use of these agents may be appropriate, even in the absence of immediate microbiological results.

This study further strengthens and deepens the evidence base for the potential future use of antiseptics in the management of equine ulcerative keratitis, extending and complementing the foundations established by previous studies [41, 43].

The results of this study show that povidone‐iodine, polyhexanide, and hypochlorous acid exhibit strong in vitro antimicrobial activity in low concentrations against *S. aureus* , *S. equi zooepidemicus* , *E. hormaechei* , and *B. cereus* , which are associated with equine ulcerative bacterial keratitis.

These agents demonstrate in vitro efficacy at concentrations well below those of commercially available formulations and those already in clinical use on the ocular surface. However, physiological factors such as tear dilution, rapid clearance, pH, and protein interference, short retention times, and surface restricted action will necessitate establishing higher concentrations for comparable antimicrobial efficacy in vivo.

## Author Contributions

**Leonie Maria Stolle:** conceptualization, project administration, methodology, investigation, formal analysis, visualization, writing – original draft, writing – review and editing, data curation. **Hilke Oltmanns:** methodology, resources, writing – review and editing, supervision, validation. **Jessica Meißner:** writing – review and editing, methodology, resources, supervision. **Frederik Heun:** investigation, writing – review and editing, methodology, conceptualization, data curation. **Ann‐Kathrin Schieder:** investigation, data curation, writing – review and editing. **Hinrich Tönjes Wolff:** investigation, methodology, conceptualization. **Bernhard Ohnesorge:** resources, supervision, project administration, conceptualization, writing – review and editing. **Claudia Busse:** project administration, writing – review and editing, conceptualization, supervision.

## Disclosure

*Artificial Intelligence Generated Content*: The authors have not used AI to generate any part of the manuscript.

## Ethics Statement

The authors have nothing to report.

## Conflicts of Interest

The authors declare no conflicts of interest.

## Data Availability

The data that support the findings of this study are available from the corresponding author upon reasonable request.
